# An Uncommon Overlap Syndrome Between Ankylosing Spondylitis and Amyotrophic Lateral Sclerosis—Case Report

**DOI:** 10.3390/medicina60101703

**Published:** 2024-10-17

**Authors:** Christian Banciu, Sorin Chiriac, Cristina Pojoga, Loredana Marian, Antonio Fabian, Armand Gogulescu, Mihaela Simu, Ramona Parvanescu, Alexandra Mioc, Roxana Racoviceanu, Andreea Munteanu

**Affiliations:** 1 Department of Internal Medicine IV, Faculty of Medicine, “Victor Babes” University of Medicine and Pharmacy, 2 Eftimie Murgu, 300041 Timisoara, Romania; banciu.christian@umft.ro (C.B.); munteanu.andreea@umft.ro (A.M.); 2 Department of Surgery III, Faculty of Medicine, “Victor Babes” University of Medicine and Pharmacy, 2 Eftimie Murgu, 300041 Timisoara, Romania; 3 Department of Gastroenterology, Regional Institute of Gastroenterology and Hepatology, 400162 Cluj-Napoca, Romania; cristina.pojoga@ubbcluj.ro; 4 Department Clinical Psychology and Psychotherapy, Babes-Bolyai University (UBB Med), 400015 Cluj-Napoca, Romania; 5 Department of Rheumatology, Timiş County Emergency Clinical Hospital, 300723 Timisoara, Romania; loredanamarian@yahoo.com; 6 Clinical Hospital of Infectious Diseases and Pneumophysiology Dr. Victor Babeș Timișoara, 300310 Timisoara, Romania; antoniofabian9319@yahoo.com; 7 Department XVI: Balneology, Medical Rehabilitation and Rheumatology, “Victor Babes” University of Medicine and Pharmacy, 2 Eftimie Murgu, 300041 Timisoara, Romania; gogulescu.armand@umft.ro; 8 Department of Neurology II, “Victor Babes” University of Medicine and Pharmacy, 2 Eftimie Murgu, 300041 Timisoara, Romania; simu.mihaela@umft.ro; 9 Department of Pharmaceutical Chemistry, “Victor Babes” University of Medicine and Pharmacy, 2 Eftimie Murgu, 300041 Timisoara, Romania; ramona.parvanescu@umft.ro (R.P.); babuta.roxana@umft.ro (R.R.); 10 Department of Pharmacology—Pharmacotherapy, Faculty of Pharmacy, “Victor Babes” University of Medicine and Pharmacy, 2 Eftimie Murgu, 300041 Timisoara, Romania; alexandra.mioc@umft.ro

**Keywords:** overlap syndrome, ankylosing spondylitis, amyotrophic lateral sclerosis, TNF-α inhibitors

## Abstract

This case report describes an uncommon overlap syndrome between ankylosing spondylitis (AS) and amyotrophic lateral sclerosis (ALS). Initially, the patient was diagnosed with AS, for which he received various specific treatments, including TNF-α inhibitors. After five years of treatment with TNF-α inhibitor etanercept, the patient was referred for a full neurological assessment after he reported balance disturbances, postural instability, muscle weakness, and other neurological symptoms that indicated the presence of a neurological disorder. After a thorough investigation, the patient was diagnosed with ALS. This case report aims to contribute to the limited literature by providing a detailed case study regarding the crosstalk between AS and ALS while also exploring the potential underlying mechanisms and the possible link between TNF-α inhibitors therapy and ALS.

## 1. Introduction

Ankylosing spondylitis (AS) is an autoimmune spondyloarthropathy involving spine and sacroiliac joints as well as their adjacent tendons and ligaments; it belongs to a larger group of spondyloarthropathies with common clinical and genetic characteristics and in severe cases may lead to fibrosis and calcification ultimately causing the loss of flexibility of the spine. The disease also involves the inflammation of the hips, shoulder, and peripheral joints and may cause extraarticular manifestations such as inflammatory bowel disease or psoriasis [1].

Neurological complications cannot be seen often in AS patients; however, they have been described as root lesions (radiculopathies), cauda equina syndrome, myelopathies (compression of the spinal cord), and myopathies [2]. The primary underlying mechanisms involved in AS nervous system co-morbidities are compression and inflammation, followed by arteritis and demyelination [2]. The inflammation triggers a consecutive repair process in which the soft tissue is ossified and fused, thus leading to bone outgrowths. With the progression of the disease, the overgrowth of the bone can lead to spinal stenosis and a fragile, stiff spine that can result in fractures, even in minor accidents [2]. Moreover, in AS patients, the inflammatory tissue and the structural changes of the spinal canal can also lead to compression and cord and nerve impingement [2]. Focal neurological manifestations may be accompanied by cognitive deficits, presumably due to the neurodegeneration induced by systemic inflammation; also, it has been postulated that the long-term administration of anti-inflammatory drugs may affect the volume of the hippocampus, which can be associated with cognitive deficit [3]. A similar observation was reported in a population-based study that reported a higher risk of dementia in people with AS [4]. A recent study demonstrated the presence of various brain structural and functional abnormalities associated with pain and fatigue in the brain of AS patients, as well as changes that were related to BASDAI and inflammation levels [5]. Although they are not frequent, neurological disorders have been associated with AS; such disorders include multiple sclerosis (MS) [6], although the two could be separate, non-related conditions, focal epilepsy and vertebrobasilar insufficiency [7]. Despite a limited number of studies and cases where AS is associated with MS, a common factor in both AS and MS pathogeny seems to be dysregulated T-cell activity, specifically the Th17 subpopulation. This subset of lymphocytes increases the production of IL-17 and can cross the blood-brain barrier, disrupting it by inducing the activation of various inflammatory cells in the central nervous system [8]. In recent years, studies have shown the involvement of the Th17/IL-17 pathway in the pathogenesis of both AS and MS [9,10,11]; positive results were obtained after the treatment with secukinumab, a monoclonal antibody that inhibits the activity of IL-17, in patients with both AS and MS [12].

Amyotrophic lateral sclerosis (ALS) is a neurodegenerative pathology that mainly affects the motor system but also displays extra-motor symptoms. ALS causes the loss of upper and lower neurons in the motor cortex, resulting in progressive muscle weakness, ultimately affecting respiratory muscles and causing death within 2–5 years after the onset of the symptoms; extra-motor symptoms that occur in approximately 50% of the patients include behaviour changes and difficulties in conducting daily tasks and speech disturbances [13]. A familial pattern has been revealed, but most cases of ALS appear sporadically; there is a clear overlap between ALS and frontotemporal dementia (FTD) in terms of clinical, pathological, and genetic features, the diagnosis of FTD being established in 5–25% of ALS patients and acting as a negative prognostic factor [14]. Studies reported the involvement of Th17 and IL-17 in the pathogenesis of neurodegenerative diseases, including ALS; Th17 cells and IL-17 directly promoted motor neuron degeneration, while treatment with anti-IL-17 reversed all the effects associated with IL-17 [15,16]. Consecutively, the study of IL-17 can be another key to understanding the shared immunological mechanisms between ALS and AS. Increasing evidence demonstrated the existence of another key factor in the development and progression of AS and ALS: mitochondrial dysfunction. In ALS patients, mitochondrial dysfunction seems to occur through various mechanisms, such as the reduced activity of mitochondrial complex I, alterations in mitochondrial fusion and fission processes, decreased mitochondrial membrane potential, disruptions in Ca^2+^ homeostasis, and excessive reactive oxygen species (ROS) production [17]. Although mitochondrial dysfunction is less studied in AS compared to ALS, several studies suggested that mitochondrial dysfunction and consecutive increased ROS production are mediators of AS pathogenesis [18,19,20], thus highlighting another potential link between these diseases.

Cases of AS overlapped with ALS are extremely rare in the medical literature. Although ALS is the most frequently encountered form of progressive motor neuron disease, it is still unclear if the two conditions coexist as separate conditions or if they are connected. This debate arose from the fact that the administration of TNF-α inhibitors as treatment for AS may trigger severe side effects, including central and peripheral demyelination disorders, due to the loss or decreased neuronal protection physiologically ensured by TNF-α activation [21]; however, ALS may also occur in AS patients with no history of biological treatments [22].

Here, we present the case of a patient with diagnosed AS who, after 8 years of various specific treatments, including TNF-α inhibitors, developed muscle weakness as well as other symptoms suspicious for neurological disorders. After a thorough neurological and imagistic evaluation, the patient was diagnosed with ALS based on nerve conduction velocity (NCV) test and electromyography (EMG).

## 2. Materials and Methods

**Figure 1:**
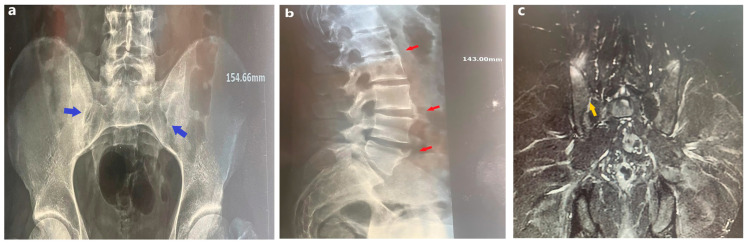
Bilateral sacroiliitis grade III and ankylosing spondylitis—anterior to posterior pelvic radiographic evaluation (**a**), lateral lumbar spine radiographic evaluation (**b**) and coronal plane MRI (**c**). Sacroiliitis with erosions, evidence of sclerosis, and an ill-defined sacroiliac joint (blue and yellow arrows). Syndesmophytes formation with a vertical orientation at the level of L1–L2 and L3–L5 (red arrows).

In 2016, a 55-year-old male patient, ex-boxer, previously diagnosed with primary arterial hypertension and type 2 diabetes mellitus, presented with symptoms of lumbar back pain lasting over three months, morning stiffness that improved with activity and did not disappear when resting. The patient evaluation also revealed a limitation of spine movement in the frontal and sagittal planes and an expansion restriction of the thoracic cage. Bilateral sacroiliitis grade III, bone oedema at the level of the sacroiliac joints and syndesmophytes at the level of L1–L2 and L3–L5 were confirmed radiographically and through magnetic resonance imaging (MRI, Siemens Magnetom Essenza, Munchen, Germany) (Figure 1).

**Figure 2:**
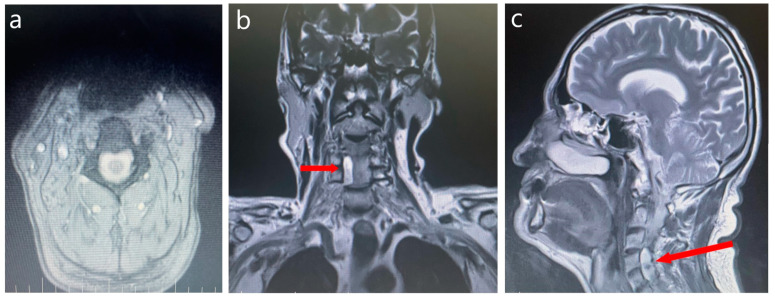
Cerebral and cervical spine MRI: axial (**a**), coronal (**b**) and sagittal (**c**) plane. The red arrows show the presence of the arachnoid cyst at the level of the C4–C5 segment.

After another 5 years, the patient sought evaluation from a neurologist, accusing a decrease in muscle strength in the upper limbs, predominantly distal right > left, that persisted for several months, balance disturbances, postural instability, and a weight decrease of 7 kg in the last month. The patient was referred to undergo cerebral and cervical spine MRI (Figure 2).

Based on the MRI results, the patient was advised to undergo further neurological evaluation for diagnostic purposes, specifically to exclude ALS. Consecutively, an NCV test and EMG (Nihon Kohden, Tokyo, Japan) were performed to assess muscular abnormalities and to confirm the suspected diagnosis.

## 3. Results

The blood test results revealed a positive expression of the human leukocyte antigen-B27 genetic marker (HLA B27), VSH = 74 mm/h, and C reactive protein (CRP) = 33 mg/mL. The Bath Ankylosing Spondylitis Disease Activity Index (BASDAI) score was 7.7. Based on the available data, the patient was diagnosed with AS, axial form. The treatment started with NSAIDs (Diclofenac 150 mg and meloxicam 15 mg) and etoricoxib (90 mg/day, when needed). The patient underwent both physiotherapy and kinesiotherapy. In January 2018, the patient started biological therapy with 50 mg Etanercept 1/week.

After another 5 years, the patient presented various neurological symptoms that prompted an evaluation from a neurologist. The results revealed the bilateral and symmetric presence of osteotendinous reflexes in the upper limbs, decreased osteotendinous reflexes in the lower limbs, a positive Babinski sign on the right part, and bilateral positive Hoffmann’s sign. The patient was assessed for motor deficits using the Medical Research Council (MRC) muscle power grade or equivalent. The results showed a tetraparesis with MRC grade 3 in the bilateral roots of the C8–T1 segment, MRC grade 4 in the bilateral C6 roots, and a tetraparesis with MRC grade 3 in the right deltoid. The local examination revealed a bilateral muscle atrophy of the upper limbs.

The RMI results confirmed the presence of a preforaminal radicular arachnoid cyst occupying the right recess of the C4–C5 segment without foraminal involvement, contrast substance uptake, and any compressive effect on the spinal cord. The spinal cord was not displaced, with no evidence of myelopathy at this level. Ischemic lesions within normal ranges were predominantly found in the cortical-subcortical frontal area. The neurological examination concluded that the neurological deficit the patient experienced was not related to the presence of the arachnoidian cyst. No surgical intervention was recommended for the arachnoid cyst as it did not compress the spinal cord or nerve roots. Also, the cerebral lesions found were not significant enough to justify the tetraparesis and the bipyramidal syndrome.

The patient was admitted to our unit for further neurological evaluation to exclude ALS and for treatment. Upon submission, the patient complained of gait and balance disturbances, frequent falls, impossibility of maintaining a prolonged orthostatic position, urinary incontinence, dysphonia, muscular atrophy with decreased muscle strength in both upper and lower limbs, loss of dexterity, inability to climb or descend the stairs alone, articular and dorsal pain. Some of these symptoms occurred after the initial neurological evaluation and have a progressive evolution towards a continuous aggravation. The hospital neurological evaluation revealed a conscious, cooperative, temporal-spatial-oriented patient; absent particular attitudes and involuntary movements; unsafe walking, with difficulty on toes, heels, and tandem; the Romberg test with unsystematised oscillations; tetraparesis upper limb: proximal right MRC + 3/5, proximal left MRC 4/5, distal right and left MRC 3/5; bilateral tetraparesis lower limbs: proximal MRC −4/5, distal MRC 4/5; mild muscle spasticity; muscular atrophies in the lower and upper limbs; good coordination revealed by the index-nose and heel–knee tests; absent patellar (PTR) and achillean (ATR) osteotendinous reflexes; present bicipital and tricipital reflexes; bilateral absent cutaneous-plantar reflex; joint pain in the lower limbs, upper limbs and spine, dysphonia, with swallowing difficulties, dysarthric speech. The Barthel Index for Activities of Daily Living score was 50, indicating a severe dependency in performing a task or activity. Based on the clinical data, the presence of ALS was suspected. The results of the NCV test and EMG revealed no alteration of the sensitive function; however, the bilateral motor function of the lower and upper limbs was significantly impaired (Table 1 and Table 2). Slowed motor nerve conduction velocity, decreased compound muscle action potential (CMAP), diffuse fasciculation, positive sharp waves, prolonged distal motor latency, increased motor unit potential (MUP), and consistent fibrillation were present and confirmed the diagnosis of sporadic ALS.

At discharge, the medical recommendations included periodic neurological follow-ups with NCV/EMG test along with a rheumatological follow-up, physiotherapy, and pharmacological treatment with riluzole (50 mg) 1-0-1, etanercept (50 mg) 1/week, *Ginkgo biloba* extract (40 mg)/day, vitamin B1 derivative and vitamin B6 (300 mg) 1-0-0, trospium chloride (30 mg) 1-0-0, atorvastatin (20 mg) 1-0-0 and esomeprazole (20 mg) 1-0-0.

## 4. Discussion

The overlap syndrome in rheumatology refers to the coexistence of two or more autoimmune connective tissue disorders [23]; autoimmune rheumatic diseases, either alone or combined in an overlap syndrome, may display central or peripheral nervous system involvement due to common autoimmune mechanisms [24]. However, autoimmune rheumatic disease displaying pure motor neuron involvement, clearly diagnosed ALS or other motor neuron pathologies, has rarely been reported in the literature. This aspect is described in a recent publication [25], where only 13 cases of ALS overlapping with rheumatoid arthritis, systemic lupus erythematosus, and Sjogren’s disease were found after a thorough literature search.

The vast majority of reported disease overlap involving ALS consisted of the simultaneous presence of multiple sclerosis [26], dementia [27], and myasthenia gravis [28]. Studies show that most ALS-identified genes are pleiotropic, thus causing multiple disease phenotypes; ALS shares genes with other neuromuscular or neurodegenerative conditions such as dementia, spinal muscular atrophy, myopathy, Parkinsonism, ataxia, neuropathy, hereditary spastic paraplegia, primary lateral sclerosis [29].

In turn, the overlap of rheumatic diseases and neurological diseases relies on autoantibodies, cytokines, chemokines, and factors that cause blood-brain barrier (BBB) dysfunction without a clearly identified mechanism. Apparently, the inflammation of the BBB facilitates the transfer of autoantibodies into the cerebrospinal fluid, which seems to be the main cause of central nervous system involvement in rheumatic diseases. Additionally, autoimmune vasculopathy and coagulopathy may be contributing factors [25].

The reflexes examination played an important role in patient evaluation, as the neurological findings offered valuable information regarding the degree of upper motor neuron (UMN) and lower motor neuron (LMN) alterations. The positive Babinski sign on the right side and the bilateral positive Hoffmann’s sign suggested damage to the corticospinal tract, indicative of underlying UMN damage. The presence of bicipital and tricipital reflexes revealed that the degeneration of UMN has not yet significantly affected the arms. However, the decreased osteotendinous reflexes in the lower limbs, absent PTR, absent ATR, and the bilateral absent cutaneous-plantar reflex suggested the presence of severe LMN deterioration. These observations are consistent with ALS, where the clinical presentation of the patient is indeed a combination of upper motor signs and lower motor neuron signs, and in our case, also explained the muscle atrophy, inability to climb or descend the stairs alone, unsafe walking, areflexia and other signs and symptoms encountered when the patient was admitted to our unit.

Cases of overlapped AS and ALS are rarely reported in the literature; to the best of our knowledge, only three cases were previously published, and, as a rule, the occurrence of neurological disorders in AS patients was not definitely linked to the evolution of the disease. The first case, reported by Loustau et al. in 2009, presented a 57-year-old patient diagnosed with ankylosing spondylitis who, due to resistance to non-steroidal anti-inflammatory drugs, was started on treatment with the TNF-α inhibitor infliximab. After 1 month of treatment, the patient developed symptoms consistent with amyotrophic lateral sclerosis, which was confirmed following an electrophysiological assessment [30]. The case presentation raised the issue of a potential causality relationship between the development of ALS and the TNF-α induced inhibition due to the fact that in ALS cases, a clear TNF-α activation was reported presumably as a neuroprotective mechanism.

A second case reported a 44-year-old AS patient who was initially diagnosed with cervical myelopathy caused by intraspinal ligament ossification, but then a final ALS diagnosis was established based on electroneuromyography [31]. As the authors pointed out, in rheumatic diseases, including AS, a series of neurological and musculoskeletal symptoms may occur, creating confusion with neurological diseases and delaying proper diagnosis; the study could not clarify if ALS developed as a complication of AS. However, in this case, the patient had not received TNF-α inhibitors; therefore, ALS cannot be regarded as a drug therapy complication. The patient was treated irregularly with sulfasalazine, which acts as a ferroptosis inducer by inhibiting the xCT system; ferroptosis is a particular iron-dependent type of programmed cell death due to lipid peroxidation which was described in most neurodegenerative diseases together with dysregulated iron homeostasis and decreased glutathione [32]. Since ferroptosis inhibitors were revealed as neuroprotectors in animal models as well as clinical trials and suggested as treatment against various neurodegenerative disorders, including ALS, one may state that sulfasalazine may cause neuronal damage and indirectly contribute to the development of neural disorders. Controversially, sulfasalazine was reported as a neuroprotector in a transient cerebral and retinal ischemia rat model where its systemic administration reduced neuronal death; the underlying mechanism consisted of the blockage of NMDA receptors and subsequent prevention of Ca ions influx and accumulation [33]. Therefore, sulfasalazine was suggested as a potential therapeutic agent against neurodegenerative diseases. These controversial findings make it impossible to designate sulfasalazine as either neuroprotector or neuro-damaging in that particular patient which supports the conclusion that ALS cannot be regarded as a complication of drug therapy.

The third case described a 39 years old patient who had been diagnosed with AS at the age of 31, but the authors failed to indicate the recommended treatment, if any; 8 years after the onset of the disease, the patient complained of muscle weakness, speech difficulties and occasional choking which indicated motor neuron disease [22]. Following clinical and radiological examination, as well as electromyography and nerve conduction velocity study, he was diagnosed with amyotrophic lateral sclerosis, the most common form of motor neuron disease. In this particular case, due to the lack of provided information, one cannot establish a potential link between ALS and drug therapy; however, the question remains if ALS occurs as a separate condition or consequent to AS complications.

In our case, the patient received biological therapy with etanercept, a TNF-α inhibitor, for the last 5 years as a treatment against AS. TNF-α inhibitors have been suggested by several studies as responsible for the potential development of neurodegenerative co-morbidities when used as therapeutic agents in rheumatic diseases [31]. TNF-α is an immune cytokine that targets two different receptors and activates various signalling cascades, triggering cellular responses depending on its molecular configuration and concentration; these complex mechanisms provide TNF-α with antagonistic effects in the central nervous system, the cytokine promoting both neuroprotective and neurotoxic, inflammatory activities [34]. As a result, TNF-α inhibitors can be successfully used as a therapy against autoimmune inflammatory diseases, where they are more effective than disease-modifying drugs but proved detrimental against neurodegenerative disorders such as multiple sclerosis. The literature reports several cases where the use of TNF-α inhibitors caused demyelination in the central and peripheral nervous system, but the exact underlying mechanism could not be established [35]. A safer therapeutic approach could involve TNF-α inhibitors that specifically target TNF receptor-1 and avoid or even stimulate TNF receptor-2 due to the latter’s antagonistic effects on TNF receptor-1. A study conducted in 2019 assessed a potential link between the administration of TNF-α inhibitors for various periods (12-120 months) and the risk of ALS [36]; the study revealed that the first symptoms of ALS occurred after 12 to 18 months of treatment with TNF-α inhibitors with a strong association between drug exposure and ALS development. Consequently, the authors suggested therapy with TNF-α inhibitors is an ALS risk factor that should be avoided when neurological signs occur or in patients with known risks such as family predisposition. Etanercept is a non-selective TNF-α inhibitor that raised suspicions of neurological adverse effects. However, a study revealed that out of 75 patients receiving TNF-α inhibitor therapy, only three developed neurological side effects, and none of them was treated with etanercept [37]. Moreover, the early administration of etanercept in a rat model of spinal cord injury dramatically reduced neuronal damage by decreasing both tissue and blood levels of inflammatory cytokines while stimulating antioxidative enzymes [38]. Similar results were reported on in vitro and in vivo models of Alzheimer’s dementia where etanercept treatment reduced the concentration of pro-inflammatory cytokines and, subsequently, neuron injury by activating the c-Jun N-terminal kinase (JNK) and nuclear factor-κB (NF-κB) pathways thus exerting neuroprotectors effects. A possible explanation is that etanercept, in addition to binding to both TNF-α receptors, also binds to lymphotoxin LTα3, which exhibits a similar structure with the soluble form of TNF-α (sTNF), considered the active form, which displays a higher affinity for TNF-α receptor-1 and mediates chronic inflammation [35]; in light of these findings, it was concluded that the selective inhibition of sTNF ensures the lack of neuronal adverse effects. Controversially, etanercept treatments were reported to cause demyelination events, such as the case of a patient with rheumatoid arthritis who developed transverse myelitis [39] or other cases of patients who developed multiple sclerosis [40,41,42]. However, to the best of our knowledge, no case of ALS has been reported to date in patients receiving etanercept as therapy for various diseases. Over the last decade, IL-17 inhibitors such as Ixekizumab and Secukinum have also been used for the treatment of AS, improving the clinical and biological signs of the active disease [43]. However, due to increased data regarding its safety and proven long-term efficacy, TNF-α inhibitors are favored over IL-17 inhibitors; according to the update offered by the American College of Rheumatology/Spondylitis Association in 2019, TNF inhibitors are recommended over IL-17 inhibitors as the first biologic treatment to be used [44]. Il-17 inhibitors are recommended in patients who do not respond to TNF-α inhibitors over the use of a second TNF-α inhibitor [44]. In our case, the AS was well-managed using the TNF-α inhibitors; the patient presented an Ankylosing Spondylitis Disease Activity Score (ASDAS) score of 1 and normal erythrocyte sedimentation rate (ESH) and C-reactive protein (CRP) values, indicating a low disease activity after etanercept treatment. Therefore, the patient did not meet the criteria for switching to IL-17 inhibitors.

Moreover, considering the patient’s history as a former boxer in his youth, in our case, it is pertinent to mention the potential link between repeated head injuries and the risk of developing ALS. However, published studies revealed an association between severe head injury and ALS development only when severe head trauma occurred within one year before diagnosis [45]. Furthermore, no significant association was found if the head injury occurred more than three years before the diagnosis of ALS [45], as is our case.

Studies of neurological complications of AS are quite rare in the medical literature. Khedr et al. reported in 2009 [2] that 25% of patients with AS developed neurological symptoms due to myelopathy or radiculopathy; however, the study concluded that the subclinical neurological complications are more frequent than the clinical ones in AS. Older studies revealed that in AS, the compression of the cord and spinal canal occurs due to the structural changes of the latter [31]. However, generally, neurologic involvement was reported in rheumatic conditions being considered a marker for an activated disease and significant morbidity [46]; in AS, it consists in spinal cord involvement due to compression induced by bone changes, including spinal fractures and spinal stenosis. A comprehensive review identified pain in AS as a neuroimmune process with altered brain networks that heightened the neurological mechanisms of pain [47]; in particular, the fatigue reported in AS appears to be associated with abnormalities within the sensorimotor network in the brain, which were also strongly linked to ALS where the altered functional connections may contribute to specific symptoms of the neurodegenerative disease [48]. Neurological symptoms in AS include neuropathic pain with numbness and pricking sensations [49] that also occur in neurodegenerative diseases such as ALS, further complicating the diagnosis. Medical professionals should be aware of the possibility of concurrent neurological illnesses and should ensure precise and accurate evaluations and diagnoses.

## 5. Conclusions and Future Perspectives

The case presented in the current paper describes the rare overlap of an axial form of AS with ALS in a patient previously treated with the TNF-α inhibitor etanercept. The patient was diagnosed with ALS by means of an NCV test and EMG. A short review of the literature aimed to identify similar cases was conducted, and the collected data were assessed to determine whether ALS developed as an AS complication or is the result of the biological therapy with etanercept. The results are controversial, with arguments for both theories; on the one hand, the neurological involvement found in AS shows some resemblance with the symptoms reported in ALS, the pain revealing both an inflammatory and a neuropathic component. On the other hand, etanercept was revealed to have a complex effect on the central nervous system, exerting simultaneously a neuroprotector as well as neurotoxic activity due to its non-selective inhibition of the TNF-α receptors. As other authors concluded as well, the potential occurrence of ALS following a previous AS diagnosis should drive medical professionals to investigate the possibility of an ALS diagnosis in AS patients complaining of severe neurological symptoms. A second conclusion is a strong possibility for etanercept to cause demyelination events as a result of its TNF-α inhibition effect, which may trigger neurodegenerative diseases such as ALS. Future anti-inflammatory therapies of AS should focus on the administration of TNF-α selective inhibitors that target only the TNF receptor-1 or sTNF in order to reduce the risk of neurodegeneration. Also, the use of TNF-α inhibitors should be limited to cases of AS without a family history of autoimmune diseases that might be more susceptible to central nervous system demyelination.

## Figures and Tables

**Table 1:** Sensory and motor nerve conduction results.

**Sensory Nerve Conduction**		
	**Velocity (m/s)**	**Amplitude (μV)**
R Median Digit III	54.5	31.5
L Median Digit III	56.7	38.4
R Sural	53.2	12.8
L Sural	52.8	10.7
**Motor Nerve Conduction**		
	**Velocity (m/s)**	**Amplitude (mV)**
R Median nerve	45.3	2.9
L Median nerve	43.5	3.1
R Ulnar	42.1	3.7
L Ulnar	44.4	3.5
R Tibial	38.5	4.8
R Peroneal	41.4	3.6

**Table 2:** Needle EMG results.

Needle EMG					
	Amplitude	Duration	Fibrillation	Fasciculation	Positive Sharp Waves
R Biceps brachii	++	Normal	−	−	−
R Triceps	++	+	−	+	+
L Abductor pollicis brevis	++	++	+	−	++
L Abductor digiti minimi	++	++	−	−	+
R Deltoid	++	Normal	−	−	−
L Cervical Paraspinal	++	+	−	−	−
L Gastrocnemius	++	+	+	++	+
L Rectus femoris	++	+	+	+	−
R Tibial	++	+	+		−

## Data Availability

The original contributions presented in the study are included in the article. Further inquiries can be directed to the corresponding author.

## References

[1] Zhu W., He X., Cheng K., Zhang L., Chen D., Wang X., Qiu G., Cao X., Weng X. (2019). Ankylosing spondylitis: Etiology, pathogenesis, and treatments. Bone Res..

[2] Khedr E.M., Rashad S.M., Hamed S.A., El-Zharaa F., Abdalla A.K.H. (2009). Neurological complications of ankylosing spondylitis: Neurophysiological assessment. Rheumatol. Int..

[3] Vitturi B.K., Suriano E.S., Pereira de Sousa A.B., Torigoe D.Y. (2020). Cognitive Impairment in Patients with Ankylosing Spondylitis. Can. J. Neurol. Sci./J. Can. Sci. Neurol..

[4] Chen K., Chen Y., Fan Y., Lin W., Lin W., Wang Y., Lin L., Chiou J., Wei J.C.-C. (2018). Rheumatic diseases are associated with a higher risk of dementia: A nation-wide, population-based, case-control study. Int. J. Rheum. Dis..

[5] Liu Q., Liao Z., Zhang Y., Lin C., He B., Fang L., Tu L., Zhao M., Wu X., Gu J. (2020). Pain- and Fatigue-Related Functional and Structural Changes in Ankylosing Spondylitis: An fRMI Study. Front. Med..

[6] Lourbopoulos A., Ioannidis P., Boura E., Antoniadis D., Karacostas D., Grigoriadis N. (2013). Coexistence of Multiple Sclerosis and Ankylosing Spondylitis: Report of Two Cases. Eur. Neurol..

[7] Li C., Wei X., Zou Q., Zhang Y., Yin X., Zhao J., Wang J. (2017). Cerebral functional deficits in patients with ankylosing spondylitis- an fMRI study. Brain Imaging Behav..

[8] Dos Passos G.R., Sato D.K., Becker J., Fujihara K. (2016). Th17 Cells Pathways in Multiple Sclerosis and Neuromyelitis Optica Spectrum Disorders: Pathophysiological and Therapeutic Implications. Mediat. Inflamm..

[9] Jethwa H., Bowness P. (2015). The interleukin (IL)-23/IL-17 axis in ankylosing spondylitis: New advances and potentials for treatment. Clin. Exp. Immunol..

[10] Zepp J., Wu L., Li X. (2011). IL-17 receptor signaling and T helper 17-mediated autoimmune demyelinating disease. Trends Immunol..

[11] Bowness P., Ridley A., Shaw J., Chan A.T., Wong-Baeza I., Fleming M., Cummings F., McMichael A., Kollnberger S. (2011). Th17 Cells Expressing KIR3DL2+ and Responsive to HLA-B27 Homodimers Are Increased in Ankylosing Spondylitis. J. Immunol..

[12] Eksin M.A., Erden A., Güven S.C., Armagan B., Ozdemir B., Karakas O., Omma A., Kucuksahin O. (2022). Secukinumab in the treatment of psoriatic arthritis or ankylosing spondyloarthritis with multiple sclerosis: A case series with literature review. Immunotherapy.

[13] Masrori P., Van Damme P. (2020). Amyotrophic lateral sclerosis: A clinical review. Eur. J. Neurol..

[14] Cividini C., Basaia S., Spinelli E.G., Canu E., Castelnovo V., Riva N., Cecchetti G., Caso F., Magnani G., Falini A. (2022). Amyotrophic Lateral Sclerosis–Frontotemporal Dementia. Neurology.

[15] Fu J., Huang Y., Bao T., Liu C., Liu X., Chen X. (2022). The role of Th17 cells/IL-17A in AD, PD, ALS and the strategic therapy targeting on IL-17A. J. Neuroinflammation.

[16] Fiala M., Chattopadhay M., La Cava A., Tse E., Liu G., Lourenco E., Eskin A., Liu P.T., Magpantay L., Tse S. (2010). IL-17A is increased in the serum and in spinal cord CD8 and mast cells of ALS patients. J. Neuroinflammation.

[17] Zhao J., Wang X., Huo Z., Chen Y., Liu J., Zhao Z., Meng F., Su Q., Bao W., Zhang L. (2022). The Impact of Mitochondrial Dysfunction in Amyotrophic Lateral Sclerosis. Cells.

[18] Sheu S.-Y., Tsuang Y.-H., Hsu F.-L., Lu F.-J., Chiang H.-C. (1997). Superoxide Anion Scavenge Effect of Quercs glauca Thunb. in Whole Blood of Patients with Ankylosing Spondylitis. Am. J. Chin. Med..

[19] Ho K.-J. (2000). The oxidative metabolism of circulating phagocytes in ankylosing spondylitis: Determination by whole blood chemiluminescence. Ann. Rheum. Dis..

[20] Ye G., Xie Z., Zeng H., Wang P., Li J., Zheng G., Wang S., Cao Q., Li M., Liu W. (2020). Oxidative stress-mediated mitochondrial dysfunction facilitates mesenchymal stem cell senescence in ankylosing spondylitis. Cell Death Dis..

[21] Börjesson A., Grundmark B., Olaisson H., Waldenlind L. (2013). Is there a link between amyotrophic lateral sclerosis and treatment with TNF-alpha inhibitors?. Ups. J. Med. Sci..

[22] Roy B., Sengupta S., Ghosh K., Mukhopadhyay S., Ghosh B. (2018). A curious case of ankylosing spondylosis and motor neuron disease: A mere coincidence or correlation?. Int. J. Appl. Basic Med. Res..

[23] Mohammed M.J., Hashim H.T., Al-Obaidi A.D., Al Shammari A. (2023). A novel overlap syndrome: Rheumatoid arthritis, Sjogren’s syndrome, antiphospholipid syndrome, and dermatomyositis. Clin. Case Reports.

[24] Vieira R.M., do Nascimento F.B.P., Barbosa Júnior A.A., Pereira I.C.M.R., Sachetto Z., Appenzeller S., Reis F. (2018). Spectrum of central nervous system involvement in rheumatic diseases: Pictorial essay. Radiol. Bras..

[25] Atalar E., Yurdakul F.G., Gök K., Güler T., Erten Ş., Yaşar E., Bodur H. (2022). Motor neuron disease in a patient with overlap syndrome (rheumatoid arthritis; systemic lupus erythematosus, Sjogren’s syndrome). Rheumatol. Int..

[26] Trojsi F., Sagnelli A., Cirillo G., Piccirillo G., Femiano C., Izzo F., Monsurrò M.R., Tedeschi G. (2012). Amyotrophic Lateral Sclerosis and Multiple Sclerosis Overlap: A Case Report. Case Rep. Med..

[27] Abramzon Y.A., Fratta P., Traynor B.J., Chia R. (2020). The Overlapping Genetics of Amyotrophic Lateral Sclerosis and Frontotemporal Dementia. Front. Neurosci..

[28] Hodzic R., Piric N., Zukic S., Cickusic A. (2021). Coexistence of myasthenia gravis and amyotrophic lateral sclerosis in a Bosnian male: An unusual clinical presentation. Acta Myol. Myopathies Cardiomyopathies Off. J. Mediterr. Soc. Myol..

[29] Olsen C.G., Busk Ø.L., Holla Ø.L., Tveten K., Holmøy T., Tysnes O.-B., Høyer H. (2024). Genetic overlap between ALS and other neurodegenerative or neuromuscular disorders. Amyotroph. Lateral Scler. Front. Degener..

[30] Loustau V., Foltz V., Poulain C., Rozenberg S., Bruneteau G. (2009). Diagnosis of amyotrophic lateral sclerosis in a patient treated with TNFα blockers for ankylosing spondylitis: Fortuitus association or new side effect of TNFα blockers?. Jt. Bone Spine.

[31] Danaci A., Dülgeroğlu D., Ünlü E., Bal A., Karaahmet Ö.Z., Çakci F.A. (2017). Coexistence of Ankylosing Spondylitis and Amyotrophic Lateral Sclerosis: Case Report. J. Phys. Med. Rehabil..

[32] Moujalled D., Strasser A., Liddell J.R. (2021). Molecular mechanisms of cell death in neurological diseases. Cell Death Differ..

[33] Ryu B.R., Lee Y.A., Won S.J., Noh J.-H., Chang S.-Y., Chung J.-M., Choi J.S., Joo C.K., Yoon S.H., Gwag B.J. (2003). The Novel Neuroprotective Action of Sulfasalazine through Blockade of NMDA Receptors. J. Pharmacol. Exp. Ther..

[34] Gonzalez Caldito N. (2023). Role of tumor necrosis factor-alpha in the central nervous system: A focus on autoimmune disorders. Front. Immunol..

[35] Kemanetzoglou E., Andreadou E. (2017). CNS Demyelination with TNF-α Blockers. Curr. Neurol. Neurosci. Rep..

[36] Petitpain N., Devos D., Bagheri H., Rocher F., Gouraud A., Masmoudi K., Coquerel A. (2019). Is <scp>TNF</scp> inhibitor exposure a risk factor for amyotrophic lateral sclerosis?. Fundam. Clin. Pharmacol..

[37] Kaltsonoudis E., Zikou A.K., Voulgari P.V., Konitsiotis S., Argyropoulou M.I., Drosos A.A. (2014). Neurological adverse events in patients receiving anti-TNF therapy: A prospective imaging and electrophysiological study. Arthritis Res. Ther..

[38] Hasturk A., Baran C., Yilmaz E., Arikan M., Togral G., Hayirli N., Erguder B., Evirgen O. (2018). Etanercept prevents histopathological damage after spinal cord injury in rats. Asian J. Neurosurg..

[39] Defty H., Sames E., Doherty T., Hughes R. (2013). Case Report of Transverse Myelitis in a Patient Receiving Etanercept for Rheumatoid Arthritis. Case Rep. Rheumatol..

[40] Pfueller C.F., Seipelt E., Zipp F., Paul F. (2008). Multiple sclerosis following etanercept treatment for ankylosing spondylitis. Scand. J. Rheumatol..

[41] Al Saieg N., Luzar M.J. (2006). Etanercept induced multiple sclerosis and transverse myelitis. J. Rheumatol..

[42] Gomez-Gallego M., Meca-Lallana J., Fernandez-Barreiro A. (2008). Multiple Sclerosis Onset during Etanercept Treatment. Eur. Neurol..

[43] Baeten D., Baraliakos X., Braun J., Sieper J., Emery P., van der Heijde D., McInnes I., van Laar J.M., Landewé R., Wordsworth P. (2013). Anti-interleukin-17A monoclonal antibody secukinumab in treatment of ankylosing spondylitis: A randomised, double-blind, placebo-controlled trial. Lancet.

[44] Ward M.M., Deodhar A., Gensler L.S., Dubreuil M., Yu D., Khan M.A., Haroon N., Borenstein D., Wang R., Biehl A. (2019). 2019 Update of the American College of Rheumatology/Spondylitis Association of America/Spondyloarthritis Research and Treatment Network Recommendations for the Treatment of Ankylosing Spondylitis and Nonradiographic Axial Spondyloarthritis. Arthritis Care Res..

[45] Peters T.L., Fang F., Weibull C.E., Sandler D.P., Kamel F., Ye W. (2013). Severe head injury and amyotrophic lateral sclerosis. Amyotroph. Lateral Scler. Front. Degener..

[46] Sofat N., Malik O., Higgens C.S. (2006). Neurological involvement in patients with rheumatic disease. QJM An Int. J. Med..

[47] Bidad K., Gracey E., Hemington K.S., Mapplebeck J.C.S., Davis K.D., Inman R.D. (2017). Pain in ankylosing spondylitis: A neuro-immune collaboration. Nat. Rev. Rheumatol..

[48] Seki S., Kitaoka Y., Kawata S., Nishiura A., Uchihashi T., Hiraoka S., Yokota Y., Isomura E.T., Kogo M., Tanaka S. (2023). Characteristics of Sensory Neuron Dysfunction in Amyotrophic Lateral Sclerosis (ALS): Potential for ALS Therapy. Biomedicines.

[49] Zhou L., Li T., Wu X., Lu H., Lin L., Ye L., Yin J., Zhao J., Wang X., Bian J. (2021). Assessment of Neuropathic Pain in Ankylosing Spondylitis: Prevalence and Characteristics. Pain Ther..

